# Using a stair horizontal-vertical illusion to increase foot clearance over an inconsistently taller stair-riser

**DOI:** 10.1371/journal.pone.0257159

**Published:** 2021-09-14

**Authors:** Timmion K. Skervin, Neil M. Thomas, Andrew J. Schofield, Mark A. Hollands, Constantinos N. Maganaris, Thomas D. O’Brien, Vasilios Baltzopoulos, Richard J. Foster

**Affiliations:** 1 Research to Improve Stair Climbing Safety, School of Sport and Exercise Sciences, Faculty of Science, Liverpool John Moores University, Liverpool, United Kingdom; 2 School of Psychology & Aston Research Centre for Healthy Ageing, College of Health and Life Sciences, Aston University, Birmingham, United Kingdom; Toronto Rehabilitation Institute - UHN, CANADA

## Abstract

**Introduction:**

Stair falls can be caused by inconsistent stair dimensions. During ascent, inconsistently taller stair risers lead to reduced foot clearances as the inconsistency goes unnoticed. A stair horizontal-vertical illusion increases perceived riser heights and foot clearance and could offset reduced foot clearances over inconsistently taller risers, though this might impact other stair safety measures.

**Method:**

Twelve participants (age: 22 (3) years) ascended a seven-step staircase under three conditions: i) all steps consistent in riser height (consistent), ii) a 1cm increase in step 5 riser height (inconsistent) and iii) a 1cm increase in step 5 riser height, superimposed with a stair horizontal-vertical illusion (illusion). Vertical foot clearance, foot overhang, and margins of stability were assessed over step 4, 5 and 6. Perceived riser height due to the illusion was determined through a computer perception test. A One-Way Repeated Measures ANOVA compared biomechanical variables between conditions. A One Sample t test compared perceived riser height to the true height.

**Results:**

Over the inconsistent step 5, foot clearance reduced by 0.8cm compared to consistent. Illusion increased foot clearance by 1.1cm and decreased foot overhang by 4% compared to inconsistent. On step 4 the illusion led to more anterior instability compared to inconsistent. Illusion and inconsistent led to more mediolateral stability compared to consistent. The illusion increased perceived riser height by 12%.

**Discussion:**

Foot clearance reductions over inconsistently taller risers can be offset by a stair horizontal-vertical illusion. Additional benefits included a safer foot overhang and unaffected stability over the inconsistent riser. Changes to step 4 stability might have resulted from leaning forward to look at the step 5 illusion. The stair horizontal-vertical illusion could be a practical solution for inconsistently taller stair risers, where a rebuild is usually the only solution.

## 1. Introduction

Stair fall accidents often result in serious injury and in severe cases can be fatal [1]. Although stair fall risk is notably heightened for an older adult largely due to age related factors such as reduced vision or musculoskeletal function [2], stairs falls occur across the lifespan [3] and are a broad societal issue. Many factors can affect stair fall risk including frailty, risky behaviour (such as carrying objects on stairs) and poor vision [2], but several previous reports demonstrate the importance of the built environment and in particular stair dimensions in dynamic balance control and the circumstance of stair falls [4–8].

The stepping action on stairs is typically an intuitive response to the step dimensions apparent to a stair user [9]. This response is informed by the visually perceived step size [10–12] and can be fine-tuned by somatosensory feedback (i.e. when the foot lands on the stair tread) from the first few repetitions of the stepping action on a specific staircase [11, 13]. This somatosensory information results from foot contact on steps and the positional feedback from the movement of the lower limbs over each step. The interaction and adaptation of stair visual perception and somatosensory feedback will determine biomechanical characteristics such as foot clearance, which determine the safety of the stair negotiation. Foot clearance during a step up is a measure reflecting the distance of the foot from catching the step edge usually at the point of step edge crossing. Inadequate foot clearance can lead to a trip/fall on stairs and is characterised by foot clearances that are low and/or variable over a step edge [14]. Foot clearance height can adapt to the visually perceived height of a step/obstacle and somatosensory feedback [10, 11, 13]. Over a perceptually taller obstacle for example, Rhea, Rietdyk [11] found foot clearances initially increase in an obstacle crossing task due to the perceived obstacle size, but over repeated trials, foot clearance height reduces likely due to somatosensory feedback.

On stairs with many steps, foot clearance is reduced and more consistent over the mid stair portion compared to stair entrance [10, 15, 16] reflecting somatosensory learning of the step dimensions. The mid stair action is more likely driven by this information than vision [17] though this can present an issue when discrete inconsistencies in step dimensions are present mid stair as they go visually unnoticed and lead to reduced foot clearance [18]. Previous ergonomic reports note this as a stair safety hazard, [9, 19], though to our knowledge, only one recent study has evidenced this experimentally. Francksen, Ackermans [18] showed that during stair ascent, foot clearances reduced by ~0.9cm in young and older adults over a mid-stair riser that was inconsistently taller by 1cm compared to when all risers were consistent in height. Importantly, participants were unable to identify any inconsistencies in stair riser height after completing the trials. This inability to detect riser-height inconsistencies may be exacerbated on visually uniform stairs where the lack of differentiating features provide no cue for visually attending to a particular step or noticing between-step differences. Inconsistent stair dimensions are likely to go unnoticed or unreported unless a serious fall or trip event occurs, yet evidence suggests the prevalence of step inconsistencies in public are quite high. In an investigation of 80 stair fall reports, around 60% of the stairs had variance in riser height [6]. Variability in step dimensions between steps should not exceed 1% in the UK [20] while in the USA a variation of 4.8mm or greater between adjacent steps is prohibited and the difference between the largest and smallest step should not exceed 9.5mm [21]. However, these regulations apply to newly built stairs meaning old stairs may not conform to this or may have inconsistencies due to the stair degrading with age. Despite the safety risk, a rebuild of these stairs may be time consuming and costly.

**Fig 1:**
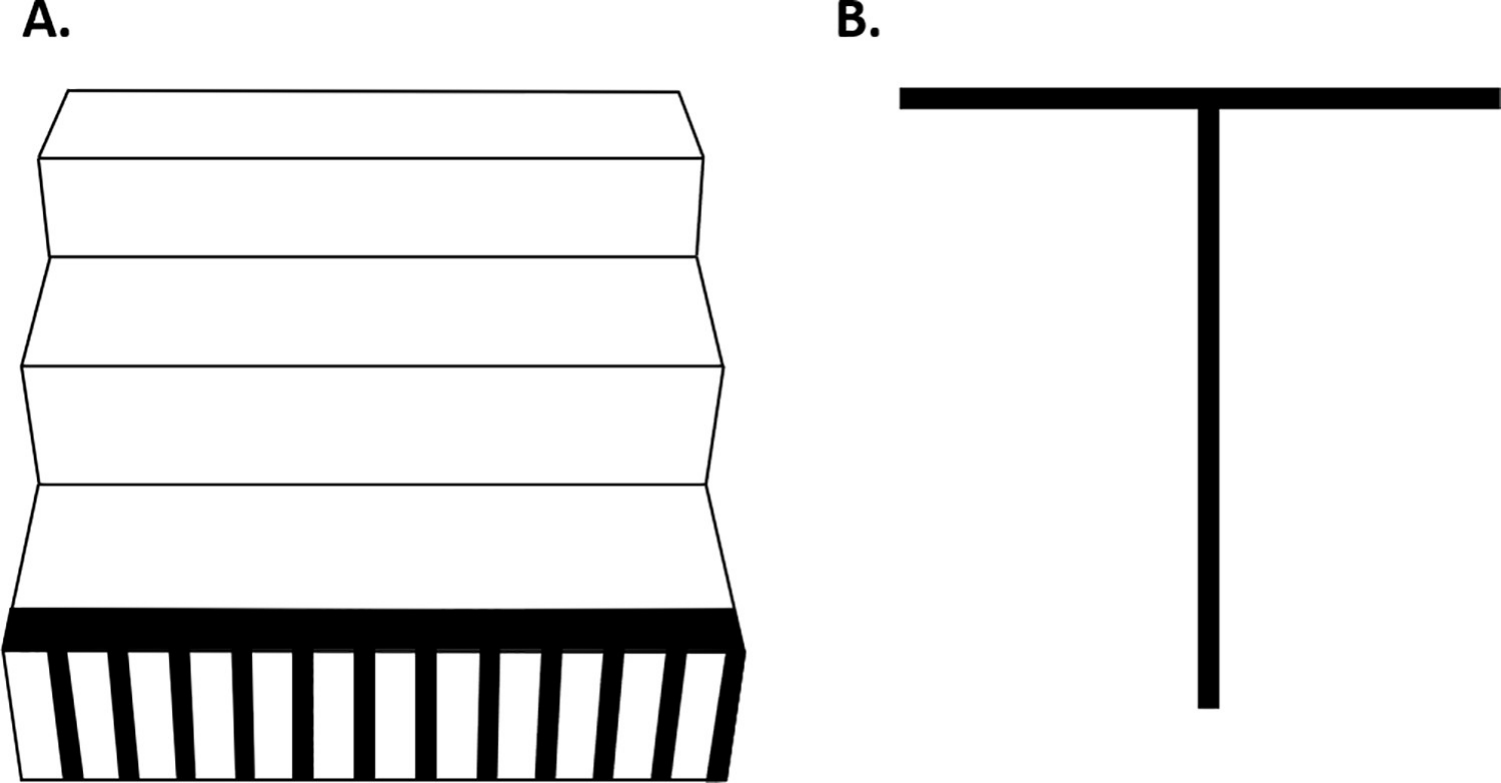
Horizontal-vertical illusions. (A) A version of the stair horizontal-vertical illusion. (B) The original horizontal-vertical illusion. The stair design is characterised by square wave gratings on the step riser and a top edge abutting edge highlighter positioned on the going of the step. The black and white stripes here are characterised by a 70–30% mark space ratio design. In the original horizontal-vertical illusion, the vertical line is perceived to be up to 20% longer than the horizontal despite both lines being of equal length.

Previous findings show that superimposing a stair horizontal-vertical (HV) illusion (Fig 1A) on to steps, stairs or obstacles can lead to subsequent increases in foot clearance of ~1cm [10, 12, 16, 22]. This visuomotor interaction is thought to represent a perception-action link [23], and the illusion was developed on the basis of the original HV illusion (Fig 1B). These findings have been demonstrated in both young and older adults [10, 16], and raise the question as to whether the stair HV illusion can ameliorate the reduced foot clearances over an inconsistently taller riser? If effective, this could be a useful solution on such stair inconsistencies as there are currently no other options to circumvent this issue other than stair rebuilds. Although previous use of the stair HV illusion has shown no detrimental effect on stair safety measures over consistent stair risers [10, 16], visually altering the perception of a riser that is physically taller than the preceding risers may pose stability issues and requires investigating.

The aim of this study was to determine whether a stair HV illusion can ameliorate the reduced foot clearances that result over an inconsistently taller mid-stair riser and to assess whether this has unintended impact on other stair ascent safety measures. These measures included foot overhang, stair balance (margins of stability) and stair velocity, which characterise other stair fall mechanisms including a stair slip or loss of balance. We hypothesised that an inconsistently taller mid-stair riser would reduce foot clearance, and that the presence of a stair HV illusion superimposed onto the inconsistently taller riser would increase foot clearance.

## 2. Materials and methods

### 2.1. Participants

Twelve young adults (Mean (1SD), age: 22 (3) years, height: 1.8 (0.1) m, mass: 81.2 (19.3) kg, visual acuity: -0.16 (0.08) LogMAR, contrast sensitivity: 2.18 (0.32) LogCS; 9 males) were recruited from the University and local community and provided written informed consent to participate. All participants were free from visual and physical/neurological impairment that would prevent them from climbing stairs. Vision was assessed using The Freiburg Visual Acuity Test [24]. All participants were naïve to the illusion and its effect from previous studies. This study received institutional ethical approval and conformed to the declaration of Helsinki.

### 2.2. Protocol

**Fig 2:**
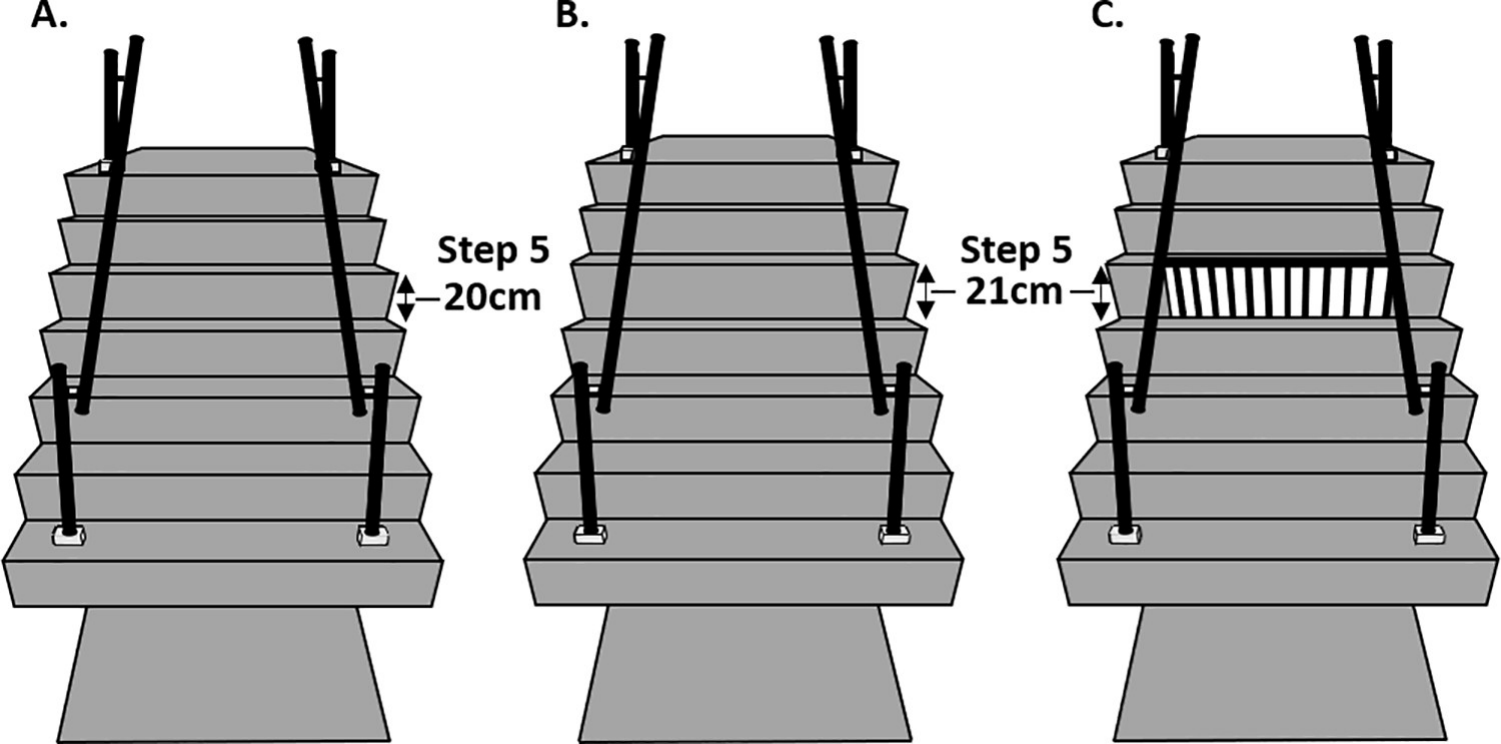
Stair conditions. (A) Consistent. (B) Inconsistent. (C) Illusion. Step 5 riser height for stair conditions B and C was increased by 1cm. The HV illusion design on stair C was characterised by a 70–30% mark space ratio between the white and black stripes on the riser aspect respectively with an abutting edge highlighter on the step going. This design was used as it showed the strongest perception-action link from our previous study [16].

Participants ascended a seven-step custom-built instrumented staircase at a self-selected speed under three different stair riser conditions: i) all seven steps consistent in riser height (consistent), ii) a 1cm increase in step 5 riser height only (inconsistent), and iii) a 1cm increase in step 5 riser height only, superimposed with a stair horizontal-vertical illusion (illusion) (Fig 2). For the consistent stair condition, each step had a riser height of 20cm. For the inconsistent and illusion stair conditions, step 5 had a rise height of 21cm whilst the remaining steps had rise heights of 20cm. All steps had a going length of 25cm irrespective of stair condition. These dimensions fall within UK building regulations [25]. Each stair condition was performed as a block of five successful trials. Participants began by completing the consistent stair condition first, but the order of inconsistent and illusion stair conditions were counterbalanced between participants. Following the stair trials, participants completed a computer-based perception test to determine the presence of a perceptual effect in response to the illusion.

Trials began approximately two/three steps away from the staircase. Participants were instructed to cross the first step with the same self-selected foot for each trial, ascend the stairs in a step-over-step manner and continue walking to the end of the top landing after crossing the last step. Upon return to the start position, participants were asked to step over two low height obstacles to disrupt any potential somatosensory interference from descending the stairs. When changing between stair conditions, participants were asked to leave the room and were instructed that something may or may not change on the stairs. Participants were not informed of the 1cm increase in riser height change or the purpose of the superimposed illusion. All participants completed the trials without handrail use. Participants wore tight clothing, flat shoes and were familiarised with the protocol. Commercially available grey floor coverings were used on the laboratory staircase to create a visually uniform stair appearance. The HV illusion riser design (Fig 2) was printed in a matte finish, cut to size, and reinforced with card. A 5.5cm edge highlighter was used on the going of step 5 to complete the illusion [10].

A 26-camera motion capture system (Vicon MX, Oxford Metrics, UK) captured whole body kinematics at 120 Hz. The Plug-in Gait marker set (without arms) was used to model head, trunk, pelvis and lower body kinematics with additional tracking markers and clusters placed on the head and lower limbs. A static calibration was captured to acquire the modelled body segments’ marker coordinates. A digitising wand (C-Motion, Germantown, MD, USA) was used to create virtual landmarks on the toe and heel-tips of participants’ shoes. Toe-tip landmarks were created on the most anterior-inferior aspect of the shoe; heel-tip landmarks were created at the most posterior-inferior aspect of the shoe. Step edge locations were defined with virtual landmarks using the digitising wand, and these were referenced to a marker cluster affixed to the stairs.

Marker data were labelled, and gap filled (quintic spline method with maximum gap of 12 frames) in Vicon (Vicon Nexus 2.6, Oxford Metrics), and exported as c3d files for analysis using Visual 3D (C-Motion, Germantown, MD, USA). Marker data were filtered using a fourth order Butterworth bidirectional filter (cut-off frequency 6Hz). Outcome measures included lead vertical foot clearance, foot overhang, margins of stability (MoS) in the anteroposterior and mediolateral directions, and stair-climbing velocity.

Lead vertical foot clearance, defined as the vertical distance of the virtual toe tip landmark to the step edge was extracted at the point where the difference in anteroposterior position between the step edge and virtual toe tip landmark was zero. Foot overhang was defined as the distance between the virtual heel tip landmark and the virtual step edge location(s) and was extracted at the point the trail limb crossed the step edge. Foot overhang was calculated as a percentage of foot length.

Centre of mass (CoM) was generated as a link model-based item in Visual 3D based on Dempster’s regression equations [26]. Stair-climbing velocity was calculated as the first derivative of the CoM anteroposterior trajectory from the start of the trial to initial contact on the top landing of the trailing foot. Margins of stability were calculated and defined in the anteroposterior direction as the distance between the extrapolated CoM (xCoM) and the virtual toe tip landmark and in the mediolateral direction as the distance between the xCoM and 5^th^ Metatarsal head [4, 27, 28]. In the anteroposterior direction a negative margin of stability value represents an xCoM anterior to the boundary of support indicating instability. In the mediolateral direction a negative value represents an xCoM that is lateral to the boundary of support indicating instability.

xCoM was defined as:

$$xCoM=pCoM+vCoM/\sqrt{\left({gl}^{-1}\right)}$$ (1)

where pCoM is the anteroposterior/mediolateral position of the CoM, vCoM is the instantaneous anteroposterior/mediolateral velocity of the CoM, *g* is acceleration due to gravity, and *l* is the absolute distance between the CoM and the ankle joint centre. The anteroposterior and mediolateral margin of stability were calculated at the point of lead vertical foot clearance over each step as this represents the most dangerous point for a trip.

Aside from stair-climbing velocity, all outcome measures were calculated for each stair condition on step 4,5 and 6 to determine whether each condition influenced behaviour before, on and after step 5.

### 2.3. Visual perception test

A computer-based perception test using a forced choice psychophysical procedure programmed in PsychoPy (Psychophysics software in Python; version 1.90) assessed the perceived height of step risers when superimposed with the stair HV illusion. This test involved the comparison of an outlined stair image superimposed with versions of HV illusions [10] on the bottom riser (fixed riser height) to plain outlined stair images with varying bottom riser heights. Participants then selected the stair that appeared to have the tallest bottom riser height over repeated trials. Perceived riser height was estimated by fitting a psychometric function to the relative step height judgments and finding the point of subjective equality between the patterned and plain steps. This test included the HV illusion that was present in the stair ascent trials (70–30% mark space ratio design; Fig 2), allowing us to understand how participants may have visually perceived the step riser height during the stair ascent trials in the illusion condition. Whilst a static perception test cannot entirely reflect visual perception dynamically during stair ascent, performing such a test with a physical set of stairs is impractical owing to the significant number of repetitive trials needed to fit a reliable psychometric function. The programming setup of the perception test included three other previously developed HV illusion designs. These designs were not included in the stair ascent assessment since no differences in perceived riser height were found between all versions (One-Way Repeated Measures ANOVA; *p>*.05). Readers are referred to [16] for our investigation of differences between illusion designs and for further details about this perception test.

### 2.4. Statistical analysis

For each participant, the average of 5 trials for each condition were used for statistical analysis. Residual plots were used to confirm normal distribution of all variables. A One-Way Repeated Measures ANOVA compared kinematic variables for within-subject effects of stair condition (x3: consistent, inconsistent, illusion). Separate ANOVAs were performed for each step. Data sphericity was assessed using Mauchly’s test of Sphericity. When data violated sphericity, a Greenhouse-Geisser (<0.75) or Huynh-Feldt (>0.75) epsilon correction was used. ANOVA effect sizes are reported as partial eta squared (n^2^_*p*_), for which the thresholds are small (0.01), medium (0.06) and large (0.14). Significant main effects were followed with post-hoc tests using a Bonferroni correction for multiple comparisons. Effect sizes for post hoc comparisons are represented as Hedges *g* for which the thresholds are small (0.2), medium (0.5) and large (0.8) [29]. A One Sample t test was used to compare the perceived riser height of the HV illusion to the true step height. Statistical analyses were performed in SPSS 26 (SPSS version 26.0 IBM Corp, 2019) with an alpha level of .05. Centre of mass data were incomplete for one participant meaning margin of stability and stair velocity comparisons were performed with eleven participants. All results are reported as mean (1SD).

## 3. Results

### 3.1. Lead vertical foot clearance

**Fig 3:**
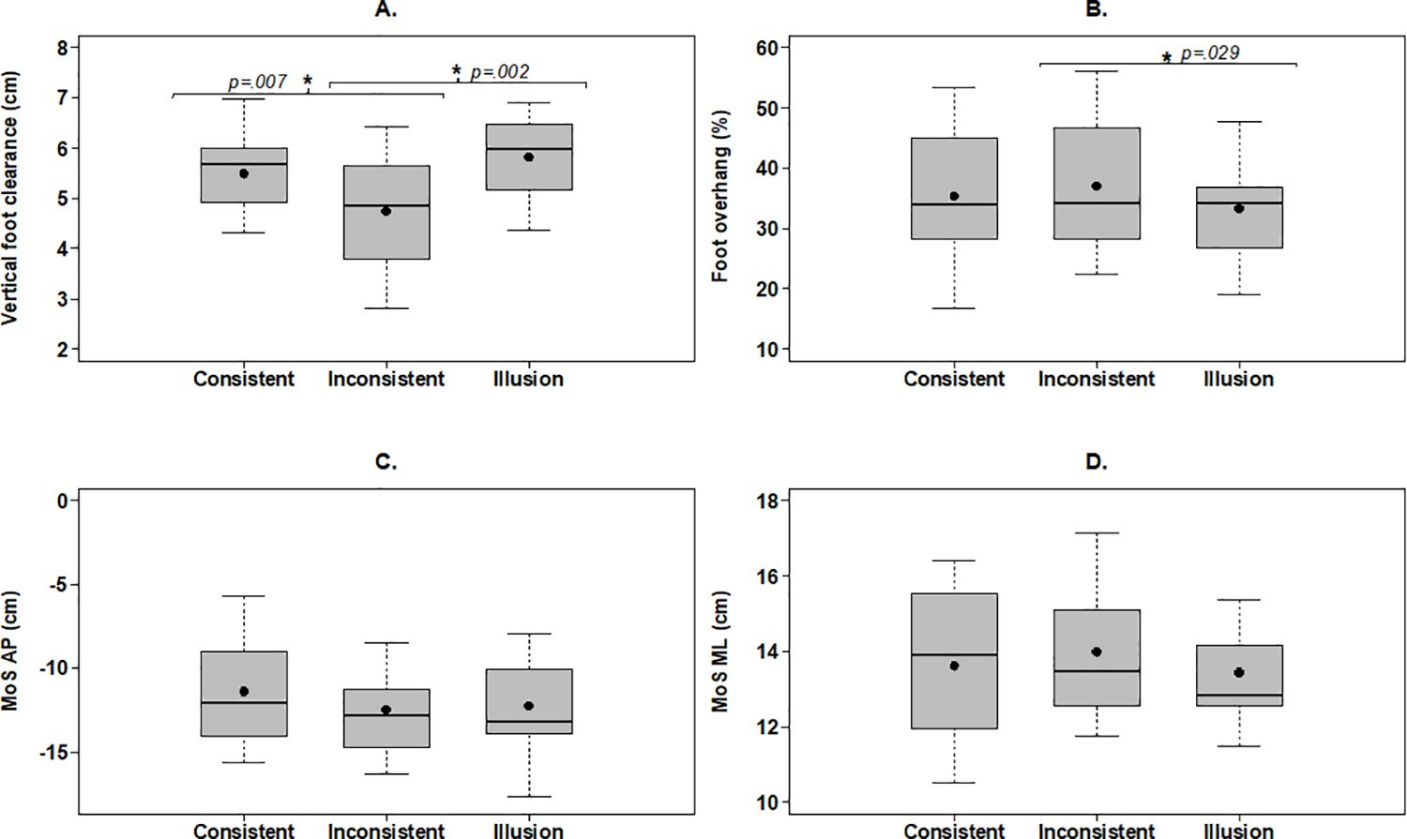
Outcome measures on step 5. (A) Vertical foot clearance. (B) Foot overhang. (C) Anteroposterior MoS. (D) Mediolateral MoS. Box plots present the mean (⬤) and median (-). *Denotes significant difference between conditions inside brackets. Foot overhang represents percentages of foot length. Negative anteroposterior MoS values represent an xCoM ahead (anterior) of the boundary of support. Positive mediolateral values represent an xCoM that is inside (medial) the boundary of support.

A significant main effect of stair condition was found over step 5 (F_(2, 22)_ = 12.413, *p = <* .001, n^2^_*p*_ = .530). The inconsistent condition reduced foot clearance by 0.8cm when compared to the consistent (*p =* .007, *g =* .*689*), and the illusion condition increased foot clearance by 1.1cm when compared to the inconsistent condition (*p =* .002, *g = 1*.*043*) (Fig 3A). No significant differences were found between the consistent and illusion condition (*p =* .615). No significant differences were found between conditions on step 4 and step 6 (Table 1; *p≥*.218).

**Table 1:** Values for vertical foot clearance, foot overhang, anteroposterior MoS and mediolateral MoS on steps 4, 5 and 6 across conditions.

	Step 4			Step 5			Step 6		
	Consistent	Inconsistent	Illusion	Consistent	Inconsistent	Illusion	Consistent	Inconsistent	Illusion
**Vertical foot**	5.0	4.7	4.9	5.5*	4.7	5.8*	4.9	4.7	5.1
**clearance (cm)**	(1.2)	(0.8)	(1.0)	(1.0)	(1.1)	(1.0)	(1.0)	(0.9)	(0.8)
**Foot overhang (%)**	39.2	41.4	40.5	35.4	37.1	33.4*	32.0	34.0	33.9
	(11.1)	(10.7)	(9.6)	(11.5)	(11.6)	(10.0)	(11.8)	(11.4)	(11.1)
**Anteroposterior MoS (cm)**	-10.0	-10.9	-11.8*	-11.4	-12.5	-12.2	-11.7	-12.9	-12.3
	(3.9)	(4.2)	(3.7)	(3.3)	(3.1)	(2.9)	(3.3)	(3.7)	(3.3)
**Mediolateral MoS (cm)**	14.2	15.1†	15.2†	13.6	14.0	13.4	14.3	15.3	14.5
	(1.8)	(2.1)	(2.2)	(2.1)	(1.8)	(1.6)	(0.9)	(1.5)	(1.7)

Negative and positive MoS values represent an xCoM ahead (anteroposterior) and inside (mediolateral) the boundary of support, respectively. Foot overhang values represent percentages of foot length.

*Denotes significant difference compared to inconsistent condition.

**†**Denotes significant difference compared to consistent condition.

### 3.2. Foot overhang

A significant main effect of stair condition was found on step 5 (F_(2, 22)_ = 4.612, *p =* .021, n^2^_*p*_ = .295). The illusion condition reduced foot overhang by 4% compared to the inconsistent condition (*p =* .029, *g =* .*327*) but was not significantly different to the consistent condition (*p =* .541) (Fig 3B). No differences were found on step 5 when the consistent condition was compared to the inconsistent condition (*p =* .387). No significant differences were found between conditions on step 4 and step 6 (*p≥*.165).

### 3.3. Anteroposterior margins of stability

For all conditions, the anteroposterior margin of stability was negative at the point of lead vertical foot clearance (Fig 3C). No significant main effect of stair condition was found on step 5 (F_(2, 20)_ = 2.391, *p =* .117, n^2^_*p*_ = .193). A significant main effect of stair condition was found on step 4 (F_(1.294, 12.941)_ = 6.288, *p =* .020, n^2^_*p*_ = .386). The illusion condition led to a more negative margin of stability when compared to the inconsistent condition by 0.9cm (*p =* .031, *g =* .*216*) but illusion was not significantly different to the consistent condition (*p =* .055). No differences were found on step 4 when comparing consistent to the inconsistent condition (*p =* .366; Table 1). A significant main effect of condition was found on step 6 (F_(2, 20)_ = 3.777, *p =* .041, n^2^_*p*_ = .274), but post hoc comparisons revealed no differences between stair conditions (*p≥*.111; see S1 Table for differences when post hoc corrections are not applied).

### 3.4. Mediolateral margins of stability

For all conditions, the mediolateral margin of stability was positive, indicating stability at the point of lead vertical foot clearance (Fig 3D). No significant main effect of condition was found on step 5 (F_(1.320, 13.203)_ = 1.231, *p =* .303, n^2^_*p*_ = .110). A significant main effect of condition was found on step 4 (F_(2, 20)_ = 7.004, *p =* .005, n^2^_*p*_ = .412). The illusion (15.2 (2.2) cm, *p =* .009) and inconsistent (15.1 (2.1) cm, *p =* .015) condition led to greater stability compared to the consistent condition (14.2 (1.8) cm; Table 1). No significant differences were found between the illusion and inconsistent condition (*p =* 1.0). A significant main effect of condition was found on step 6 (F_(2, 20)_ = 3.684, *p =* .043, n^2^_*p*_ = .269), but post hoc comparisons revealed no differences between stair conditions (*p≥*.054; see S1 Table for differences when post hoc corrections are not applied).

### 3.5. Stair-climbing velocity and perceived riser heights

Stair-climbing velocity did not significantly differ between stair conditions (consistent = 0.5 (0.1) m.s^-1^, inconsistent = 0.5 (0.1) m.s^-1^, illusion = 0.5 (0.04) m.s^-1^) (F_(3, 33)_ = .788, *p =* .509, n^2^_*p*_ = .067). In the computer-based perception test, the step superimposed with the HV illusion was perceived to be significantly taller than the true height by 12% (*p <* .001, *g = 2*.*216*).

## 4. Discussion

This study is the first to provide evidence that presence of a stair HV illusion can ameliorate the effects of previously reported reduced foot clearance over an inconsistently taller mid-stair riser.

The reduced foot clearance observed for the inconsistent stair condition (0.8cm) corroborates the previously identified stair safety issue that individuals do not adapt to such riser height increases [18], likely due to the stair riser increase going unnoticed. Here we show a reduction in foot clearance that is comparable in magnitude to the inconsistency of the stair riser and similar to the reduction (0.9cm) reported by Francksen, Ackermans [18]. Whilst Francksen, Ackermans [18] created an inconsistency on the third stair riser, our inconsistency occurred on the fifth stair riser, suggesting the lack of foot clearance adaptation we found could have been driven by somatosensory learning from as early as two complete steps up.

Foot clearance for the illusion compared to inconsistent stair condition increased, suggesting the stair HV illusion is an effective visual cue that can offset the foot clearance reductions to a safer distance. No changes to foot clearance on step 4 or step 6 across stair conditions also indicate that the foot clearance increase is pertinent to the step the illusion is placed upon, supporting previous findings [10, 16]. Our computer-based perception test also showed increases in perceived riser height in response to the same HV illusion we used during the stair ascent trials. This, alongside accompanying increases in foot clearance, represents a perception-action link. This is important as studies involving motor control in response to illusions sometimes show dissociations between the perceptual response and motor action [30], though this may also be linked to methodological factors [31].

On Step 5, the HV illusion resulted in reduced foot overhang compared to the inconsistent condition, meaning a greater portion (4%) of the foot was in contact with the step. Our effect sizes however indicate that this was a small effect. Greater foot contact length on a step reduces the likelihood of a slip occurring and may be related to the presence of the edge highlighter on the going of the step. Previous findings indicate that the presence and positioning of an edge highlighter provides a visual cue that affects where the foot is placed when descending stairs [32] and may have a similar effect for stair ascent. This greater foot contact length may also be a result from the increased foot clearance height, as the foot would likely have longer time to travel forwards and downwards onto the step.

On step 4, there was a more negative anterior margin of stability for the illusion condition compared to the inconsistent stair condition, though a negative anterior margin of stability is expected between steps as part of the natural forward movement [33] on stairs. Minimal difference in foot overhang on step 4 between conditions indicates that this change was not a result of changes to the base of support positioning (i.e., anterior foot placement) on the step and more likely a change in the CoM control. This might reflect a more forward leaning upper body posture and head flexion to visually focus on the step with the stair HV illusion, particularly as this step would appear noticeably different to the other steps. During stair descent Bosse, Oberländer [28] showed more negative anterior margin of stability at touch down and an associated increased trunk flexion angle trend which could be the case here. On step 4 a significant increase in mediolateral stability was observed in the inconsistent and illusion condition when compared to the consistent condition. Importantly, the mediolateral margin of stability was positive across all conditions indicating stability and this direction of change for the inconsistent and illusion condition was towards a safer margin of stability. The reason for this change is not clear but may be related to the step height change rather than the step appearance given the null difference between the illusion and inconsistent. Over the entire stair ascent period, no significant differences between conditions were observed for stair velocity. This suggests that the step manipulations do not significantly introduce overall stair hesitation. On step 6, differences in margin of stability did not reach statistical significance due to the post hoc correction for multiple comparisons. Our supplementary material provides an indication where differences may have occurred between conditions if statistical significance were achieved. These findings point towards reduced anteroposterior stability for inconsistent when compared to consistent, but greater mediolateral stability for inconsistent when compared to the other conditions. No differences are indicated between illusion and consistent suggesting the stair HV illusion likely does not disrupt normal stair balance over step 6.

For an inconsistent riser, the stair HV illusion may have advantages over the use of an edge highlighter alone. On a single step, edge highlighters appear not to increase foot clearance during the step up (despite the added saliency), whereas a stair HV illusion does [10]. This means on an inconsistent riser a stair HV illusion would more likely increase foot clearance compared to an edge highlighter. The stair HV illusion incorporates an edge highlighter in its design which can aid stair descent safety [32] and could be a possible solution for inconsistent goings, though future work should address this. The riser stripes alongside the top edge highlighter provides greater saliency to the step and may encourage visual attention, particularly for an inconsistent riser which goes unnoticed. The stair HV illusion could therefore be a practical solution for inconsistently taller risers on public stairs, where a rebuild is usually the only option.

### 4.1. Limitations and future considerations

Here we demonstrate a stair safety benefit (i.e., increased foot clearance) by superimposing the stair HV illusion on an inconsistently taller riser, in young adults only. Future research should determine whether older adults, who fall with more serious consequences than young adults, respond in a similar way. Francksen, Ackermans [18] showed no differences in reduced foot clearances between young and older adults over an inconsistently taller stair riser, suggesting findings from the current study may translate to older adults. Our previous work indicates a perception-action link in older adults in response to the stair HV illusion superimposed on stairs [16], so it is plausible that older adults could exhibit increases in foot clearance in response to a stair HV illusion placed onto an inconsistently taller riser in the same way as young adults in the current study. For our consistent condition we chose a plain/uniform stair surface without edge highlighters as these are not always present on stairs. Foster, Whitaker [10] showed foot clearance does not increase significantly with the presence of an edge highlighter alone, but does when a stair HV illusion is used. Here it is likely an increase in foot clearance would still occur with a stair HV illusion if all steps had edge highlighters present, though this should be tested explicitly in future. This study tested more males than females, therefore care should be taken when generalising the current findings to females since sex-based differences have been previously reported in stair negotiation. In a stair descent task, Hsue and Su [34] showed women to have larger peak to peak CoM displacement and peak instantaneous CoM velocity in the AP, ML and vertical directions compared to men which could affect dynamic stability. Our margin of stability measure was captured at the point where the foot is most likely to catch/contact the inconsistently taller stair riser. Future research could also look to assess whether differences in stability occur across other periods of the gait cycle (e.g., in a time series analysis) when a stair HV illusion is used on an inconsistently taller riser. Future investigations should also assess the efficacy of using the stair HV illusion on stairs in public where cases of inconsistently taller risers are reported. This will also help to establish whether the effectiveness of the illusion translates to real world conditions where factors such as crowds on stairs or distractions to stair visual attention might influence the overall stair safety benefit the illusion provides.

## 5. Conclusion

We provide evidence that the presence of a stair HV illusion can offset the effects of reduced foot clearance over an inconsistently taller mid-stair riser. Importantly, there were no detrimental effects on other measures of stair safety over the inconsistently taller step. The stair HV illusion could be a beneficial solution on stairs that have an inconsistent riser and future research should determine its efficacy in real-world environments with younger and older adult stair users.

## Supporting information

S1 Table Bonferroni corrected and uncorrected post hoc statistical tests for margins of stability on step 6. (DOCX)

S1 Data (XLSX)
