# Supplementary material for: Using a stair horizontal-vertical illusion to increase foot clearance over an inconsistently taller stair-riser
Source: PLoS One. 2021 Sep 14;16(9):e0257159. doi: 10.1371/journal.pone.0257159 (PMC8439448; doi:10.1371/journal.pone.0257159)
Supplement: S1 Table — (DOCX) [file pone.0257159.s001.docx]

**S1 Table.** **Bonferroni corrected and uncorrected post hoc statistical tests for margins of stability on step 6.**

| **Step 6 anteroposterior margin of stability** | | |
| --- | --- | --- |
|  | **Bonferroni corrected post hoc** | **Uncorrected post hoc** |
| Illusion vs inconsistent | *p=*.196 | *p*=.065 |
| Consistent vs inconsistent | *p=*.111 | *p*=.037 |
| Illusion vs consistent | *p=*.775 | *p*=.258 |
| **Step 6 mediolateral margin of stability** | | |
|  |  |  |
| Illusion vs inconsistent | *p=*.054 | *p*=.018 |
| Consistent vs inconsistent | *p=*.066 | *p*=.022 |
| Illusion vs consistent | *p=1*.000 | *p*=.636 |

Statistical significance achieved through uncorrected post hoc’s are shaded in grey.
